# The Long and Winding Road: Early Years of the Accreditation for Gerontology Education Council

**DOI:** 10.1093/geroni/igab046.157

**Published:** 2021-12-17

**Authors:** Robert Maiden, Donna Schafer

## Abstract

The long road of establishing an accreditation entity began in August 2010 when the AGHE Accreditation Task Force was convened. After numerous meetings complete with loud and vigorous debates, AGEC, the Accreditation for Gerontology Education Council emerged in 2016. Over the subsequent years, the Standards hit the hard road of reality leading to various revisions to the Handbook. The symposium’s first presentation concerns the history of AGEC and its further development into an independent entity. The key purpose of AGEC is assuring gerontology programs educational quality and enhancement governed by the principle of self-evaluation and peer review that engenders trust. The next presentation discusses the marketing aspect of AGEC built on getting feedback from the public. One of the outcomes of conducting focus groups and surveying the public is the discovery that prospective students really see the value of accreditation. The penultimate presentation focuses on refinements to procedures alluded to in the first presentation in response to the feedback received in meeting with institutions and faculty about what accreditation offers to students, stake-holders, and ultimately the older adults served by the graduates in the work force. The key goal is to clarify the expectations and simplify the application process. On no other issue has more time been spent than on the assessment of students’ competency. Our last presentation explains competency-based education consisting of well-articulated student learning outcome measures that are consonant with the program’s mission that lead to "closing the loop" of continuous and durable improvements in the learning environment.

